# Self-perceived stress reactivity is an indicator of psychosocial impairment at the workplace

**DOI:** 10.1186/1471-2458-10-252

**Published:** 2010-05-14

**Authors:** Heribert Limm, Peter Angerer, Mechthild Heinmueller, Birgitt Marten-Mittag, Urs M Nater, Harald Guendel

**Affiliations:** 1 Department of Psychosomatic Medicine and Psychotherapy, Technische Universitaet Muenchen (TUM), Langerstrasse 3/I, 81675 Munich, Germany; 2 Department of Occupational, Social and Environmental Medicine, Ludwig-Maximilians-University, Ziemssenstr. 1, 80336 Munich, Germany; 3 University of Zürich, Dept. of Psychology, Clinical Psychology and Psychotherapy, Binzmuehlenstrasse 14/Box 26, 8050 Zuerich, Switzerland; 4 Department of Psychosomatic Medicine and Psychotherapy, University Hospital of Ulm, Am Hochstraess 8, D-89081 Ulm, Germany

## Abstract

**Background:**

Work related stress is associated with a range of debilitating health outcomes. However, no unanimously accepted assessment tool exists for the early identification of individuals suffering from chronic job stress. The psychological concept of self-perceived stress reactivity refers to the individual disposition of a person to answer stressors with immediate as well as long lasting stress reactions, and it could be a valid indicator of current as well as prospective adverse health outcomes. The aim of this study was to determine the extent to which perceived stress reactivity correlates with various parameters of psychosocial health, cardiovascular risk factors, and parameters of chronic stress and job stress in a sample of middle-aged industrial employees in a so-called "sandwich-position".

**Methods:**

In this cross-sectional study, a total of 174 industrial employees were assessed for psychosocial and biological stress parameters. Differences between groups with high and low stress reactivity were analysed. Logistic regression models were applied to identify which parameters allow to predict perceived high versus low stress reactivity.

**Results:**

In our sample various parameters of psychosocial stress like chronic stress and effort-reward imbalance were significantly increased in comparison to the normal population. Compared to employees with perceived low stress reactivity, those with perceived high stress reactivity showed poorer results in health-related complaints, depression, anxiety, sports behaviour, chronic stress, and effort-reward imbalance. The educational status of employees with perceived low stress reactivity is higher. Education, cardiovascular complaints, chronic stress, and effort-reward imbalance were moderate predictors for perceived stress reactivity. However, no relationship was found between stress reactivity and cardiovascular risk factors in our sample.

**Conclusions:**

Job stress is a major burden in a relevant subgroup of industrial employees in a middle management position. Self-perceived stress reactivity seems to be an appropriate concept to identify employees who experience psychosocial stress and associated psychological problems at the workplace.

## Background

Organisational restructuring, downsizing, and increasing pressure on management in keeping up with performance figures have radically transformed the nature of work [1]. Chronic job stress can be associated with a range of debilitating health outcomes, including cardiovascular diseases [2,3], depression and anxiety [4-7], musculoskeletal problems [8,9], or alcohol dependence [10,11]. Stress-associated health problems at the workplace lead to approximately 50-60% of total sick leave days [12]. Within the European Union, 350 million work days are lost because of stress-related ill-health, resulting in an overall cost of at least 20 billion € each year [13]. In the United States alone, businesses loose 300 billion $ annually as a result of lowered productivity, days absent, and health-care costs due to stress [14]. Increasingly, experts call for the implementation of workplace health promotion programmes, as psychological problems, stress-related physical complaints [3,14], perceived work stress [3,14-18], and obesity [19] have negative effects on workers' health as well as overall productivity [14,20-23]. In particular, employees who suffer from chronic job stress should be identified at an early stage so that preventive action can be taken.

So far, no unanimously accepted psychometric or biological indicator exists for the early identification and evaluation of individuals suffering from chronic job stress [24-26]. In human stress theories, it is assumed that stress arises from person-environment interactions. Thus, in addition to situational factors representing stressful demands, the ability to cope with job stress is assumed to be influenced by subjective emotional and physiological reactions in different stressful situations, which has been conceptualised as stress reactivity [27]. Somatic parameters for physiological stress reactivity, like salivary cortisol, heart rate, cardiovascular responses, and arterial catecholamine reactivity are well known. But there is still a lack of self-report questionnaires focussing on subjective, self-perceived reactions to stressful demands and situations. The stress reactivity scale (SRS) [28] is a self-report questionnaire which assesses on multiple levels typical subjective reactions to different stressful situations. It refers to the disposition of a person to answer stressors with immediate, intense, and long lasting stress reactions. By focussing on the frequency of recent stress events this concept specifically reflects on environmental components with regard to the person-environment interaction in the stress process [27]. In contrast to more traditional stress concepts based on personality traits this specific focus makes the SRS more sensitive to the measurement of change. In a laboratory stress study self-perceived stress reactivity has recently been shown to be related to personality traits, bodily complaints, sleep-related variables and cortisol reactions in a dermatological patient sample. Thus, on a psychological level, perceived stress reactivity may be a valid marker for current as well as prospective adverse health outcome [27-29]. However, this hypothesis has not yet been verified within a workplace sample.

Given the very small empirical basis of self-perceived stress reactivity and the complete lack of studies in workplace samples, there is a great need to better understand the correlations and interactions between self-perceived stress reactivity and different measures of chronic stress, job stress, somatic parameters for stress, cardiovascular risk factors, and psychosocial health outcomes. Industrial employees in a middle management ("sandwich") position seem to be an especially interesting group to test for this concept [1,3].

The present study therefore presents data of a work-health promotion study in a large sample of middle-aged industrial employees with leadership responsibility in a typical middle management position of a large international manufacturing company. The primary aim of this study was to analyse the relationship of dispositional self-perceived stress reactivity with various psychological as well as biological indicators of stress and health. Therefore, we hypothesised that high stress reactive employees have a significantly worse status of health and stress-related phenomena than for low stress reactive employees (H1). The secondary aim of this study was to identify potential key variables that explain part of the observed variance in the self-perceived stress reactivity. We hypothesised that heightened stress reactivity can be predicted by psychosocial as well as biological parameters of stress and health (H2).

## Methods

### Overview of the Design, Procedures and Participants

Participants were recruited from an international manufacturing company. At the time of the study 262 employees were deemed eligible for the study. Following two information sessions for this group, a total of 189 employees agreed to participate voluntarily in the study. 15 of the 189 interested employees failed to fulfill inclusion criteria. Thus 174 employees (92.1%) were finally included into the study. All participants were invited to an 1 1/2 hour medical examination including blood sampling and were asked to provide saliva the following working day to determine the cortisol level. Moreover, the participants were required to complete an assessment battery of questionnaires. The inclusion criteria of the study were: 1) Industrial employees in the production line with leadership responsibility ("sandwich-position"); 2) age between 18 and 65 years with more than 2 years until retirement. Application to early retirement, surgery, or other severe diseases potentially leading to more than 30 sick leave days led to exclusion. Written informed consent was obtained. Information was provided according to the Declaration of Helsinki [30]. The Ethical Committee of the University of Munich approved the study.

### Measures

#### Medical diagnostics

The medical examination included questions on the medical and family history of the participants, smoking habits, consumption of alcohol, physical activity, and current medication. We determined anthropometric data [body mass index and waist circumference (cut off score: BMI > 30, waist cut off score 102 cm)], systolic and diastolic blood pressure, 24 hours ECG, saliva cortisol and alpha-amylase levels, levels of serum low-density lipoprotein (LDL), serum high-density lipoprotein (HDL), cholesterol, glycosylated hemoglobin, triglycerides, serum dehydroepiandrosterone sulfate (DHEA-S), fibrinogen, CRP, and serum creatinin. Systolic and diastolic blood pressure were measured as the average of 2 readings obtained with a digital blood pressure instrument (Boso Inc.), the waist circumference was measured in a standing position at the level of the umbilicus or, in obese persons, at the maximum between the iliac crest and the lowest rib. Blood samples for assays of HDL, cholesterol, HbA1c, and DHEA-S were obtained in the morning. All samples were immediately processed and frozen at -200 C according to standard laboratory procedures. Saliva samples were collected using cotton dental rolls held in the mouth until saturated, and then stored in Salivette tubes (Sarstedt, Germany). Participants were instructed to give 7 samples over a single working day, with measures on waking up, 30, and 60 mins later, and then within defined time intervals throughout the day and evening (08.00 am, 11.00 am, 3.00 pm and 8.00 pm). Each participant registered in a diary the exact timing when the sample was taken as well as the time of waking up. Tubes were returned to the investigators in person and salivary free cortisol was analysed using a commercial chemiluminescence immunoassay (CLIA; IBL Hamburg, Germany). Alpha-amylase was determined by using the automatic analyser Cobas Mira and assay kits obtained from Roche [31].

#### Assessment of somatic correlates of stress

The selection of biological indicators in our study was based on the allostatic stress load concept [14,32,33]. The original allostatic load score included the following variables: systolic and diastolic blood pressure, waist-hip ratio, plasma levels of cholesterol, high-density lipoproteins (HDL), and dehydroepiandrosterone-sulphate (DHEA-S), blood levels of glycosylated haemoglobin (HbA1c), and overnight urinary secretion of cortisol, epinephrine, and norepinephrine [34]. The MacArthur study investigators also encouraged to include additional or even different biological risk factors for adverse outcomes [33]. Our revised allostatic load score was calculated as the number of the following variables that were in the highest and lowest quartiles of their distribution: Highest in systolic/diastolic blood pressure, nocturnal heart rate, waist circumference, serum LDL cholesterol, glycosylated hemoglobin, triglycerides, total output of diurnal salivary cortisol, total output of diurnal salivary alpha-amylase, fibrinogen, CRP; lowest in serum HDL cholesterol and serum dehydroepiandrosterone sulfate (DHEA-S). The distribution of each biological marker was examined for outliers and symmetry.

#### Sociodemographic and job specific data

Sociodemographic data and job specific questions concerning professional status and working time were recorded.

#### Assessment of self-perceived stress reactivity, chronic stress and job stress

Self-perceived stress reactivity was measured with the 29-item-"Stress-Reactivity Scale (SRS, see sample items below)". Perceived stress reactivity is assumed to be determined by a disadvantageous pattern of personality traits. It refers to the disposition of a person to respond to stressors with immediate, intense and long lasting stress response characteristics. The SRS quantifies general stress reactivity and stress reactivity in specific domains (social conflict, social evaluation, failure at work, and work overload). Two scales evaluate stress reactivity before and after stressful events in general and a total score can be generated. A satisfying Chronbach's Alpha between .71 ≤ α ≥ .82 for the various subscales and of .91 for the of the SRS total score has been reported. In the present sample internal consistencies (Chronbach's Alpha) ranges between .65 ≤ α ≥ .78 for the various subscales and .92 for the total score. Furthermore, factorial validity and correlations with construct-related personality traits, bodily complaints, sleep behaviour, chronic diseases, and cortisol reactions have been described [28]. In addition, findings from a twin study suggest that perceived stress is in part heritable [27]. Sample questions: (1) If I have little time for my work... Answers: 1 = I mostly stay calm; 2 = I mostly get uneasy; 3 = I mostly get quite hectic. (2) When I argue with other people ... Answers: 1 = I mostly calm down fast; 2 = I mostly stay aroused for some time; 3 = It mostly takes a very long time until I calm down again. (3) When I have to speak in front of other people ... Answers: 1 = I am mostly very nervous; 2 = I am mostly slightly nervous; 3 = I generally keep my balance.

The amount of chronic stress within the last 3 months was measured by the 12-item global scale (Screening-Scale of Chronic Stress, SSCS) of the "Trier Inventory for the Assessment of Chronic Stress (TICS)" using a four-point Likert scale [35].

Job stress was assessed with the "Effort-Reward Imbalance" (ERI) which claims that failed reciprocity in terms of high efforts spent and low rewards received in turn is likely to elicit recurrent negative emotions and sustained stress responses in exposed people. Conversely, positive emotions evoked by appropriate social rewards promote well-being, health and survival. The ERI model has been operationalised as a standardised self-report measure containing 23 Likert-scaled items in its established short version. These items define three unidimensional scales: 'effort' (6 items), 'reward' (11 items), and 'overcommitment' (6 items) with each item rated on a five-point (effort, reward) Likert scale [2,15,36].

#### Assessment of self-rated health status/behaviour

To assess the current health status of the participants, the following indicators were selected.

The Giessen Subjective Complaints List (GBB-24) is a questionnaire for health-related quality of life, and assesses four domains of physical and psychosomatic complaints by means of 24 items on a five-point Likert scale, which includes heart symptoms, fatigue, stomach pain as well as arm and leg pain [37]. Additionally, the participants answered questions on their sports-, sleeping- and smoking-behaviour.

Anxiety and depression was assessed with the Hospital Anxiety and Depression Scale (HADS) [38]. This scale is a widely used instrument to measure psychological morbidity. The HADS contains 14 items and consists of two subscales: anxiety and depression. Values between 8 and 10 are judged for signs of clinical anxiety/depression with values above 10 as indicators for the need of professional treatment.

### Analyses

Means or proportions for sociodemographic and work-related characteristics, for health status and health behaviour as well as for psychosocial stress parameters were computed for the total sample (n = 174) and for two groups with low (n = 88) and high (n = 86) stress reactivity based on a median split of the SRS total score. In all between-group comparisons significance of differences in means was tested with t-tests or Mann-Whitney-U-tests, significance of differences in proportions was tested by Chi^2^-tests. Significance of differences between sample means and reference values was tested with t-tests. For the calculation of subscale values a maximum 30% missing items per subscale was accepted. To assess whether and to which extent self-perceived stress reactivity can be predicted by psychosocial and biological factors and health-related behaviour, a logistic regression model was applied with high vs. low stress reactivity as dependent variable. The criterion for independent variables to be entered into the initial model was a p-value < .250 in univariate between-group comparisons (tables 1, 2, 3, 4). All independent variables were included as categorical variables: for HADS scores we defined three categories as suggested by the authors (<8 vs. > = 8 and <11 vs. > = 11; [38]); total cholesterol was dichotomised with cut-off < = 240 mg; all other scores were trichotomised based on tertile cut-offs. Intensive sports behaviour was categorised into none vs. 1-3 hours vs. >3 hours a week. Likelihood ratio statistic was used to select variables for removal from the model by stepwise backward elimination; probability for entry and removal was p < .05. Results are reported as odds ratios and 95% confidence intervals. Statistical significance was assumed at p < . 05. Statistical analyses were conducted using SPSS (version 15.0).

**Table 1:** Demographic and professional variables of the whole sample and in the two groups of high vs. low stress reactivity^+^

Characteristic	Total (n = 174)	Group with low stress reactivity (n = 88)	Group with high stress reactivity (n = 86)	P value
**Demographic variables**				
Age (years)	40.9 (7.8)	41.3 (7.5)	40.5 (8.1)	.477 ^c^
Males	171 (98.3%)	87 (98.9%)	84 (97.7%)	.618 ^b^
Education				
Low	99 (56.9%)	44 (50.0%)	55 (64.0%)	**< .001**^**b**^
Middle	34 (19.5%)	11 (12.5%)	23 (26.7%)	
Master degree	41 (23.6%)	33 (37.5%)	8 (9.3%)	

**Professional variables**				
Professional status				
Middle Management	74 (54.4%)	34 (47.9%)	40 (61.5%)	.343 ^b^
Middle Management deputy	29 (21.3%)	18 (25.4%)	11 (16.9%)	
Team supervisors	33 (24.3%)	19 (20.8%)	14 (21.5%)	
Others	38 (21.8%)	17 (19.3%)	21 (24.4%)	
				
Shift work	109 (62.6%)	55 (62.5%)	54 (62.8%)	1.000 ^b^
Hours of work overtime per month in h (not paid)	1.9 (7.34)	2.5 (9.7)	1.4 (3.9)	.973 ^a^
Daily break time in min	36.8 (10.4)	38.0 (10.1)	35.6 (10.7)	.117 ^a^
				
Self-reported sick leave days				
0 days 1-10 days	97 (55.7%)	48 (54.5%)	49 (57.0%)	.911 b
1-10 days	55 (31.6%)	28 (31.8%)	27 (31.4%)	
Over 10 days	22 (12.6%)	12 (13.6%)	10 (11.6%)	

^+ ^= mean (SD), unless otherwise stated.

a = Mann Whitney U-test

b = Chi^2 ^test

c = t-test

**Table 2:** Parameters of health status and health behaviour in the total sample and stratified for two groups of low vs. high stress reactivity ^+^

Characteristic	Total (n = 174)	Group with low stress reactivity (n = 88)	Group with high stress reactivity (n = 86)	P value
**Health status**				
Psychosomatic total score (GBB-24)	9.45 (9.3)	7.73 (8.4)	11.22 (9.9)	**.009**^**a**^
Exhaustion	2.41 (3.6)	1.89 (2.9)	2.94 (4.2)	.300 ^a^
Gastrointestinal complaints	1.87 (2.4)	1.76 (2.7)	1.99 (2.0)	133 ^a^
Musculoskeletal complaints	3.86 (3.5)	3.31 (3.3)	4.43 (3.5)	**.020**^**a**^
Cardiovascular complaints	1.31 (2.2)	0.77 (1.7)	1.86 (2.4)	**< .001**^**a**^
				
Depression (HADS)	4.42 (3.25)	3.26 (2.8)	5.60 (3.2)	**< .001**^**a**^
Anxiety (HADS)	6.01 (3.07)	4.93 (2.9)	7.12 (2.8)	**< .001**^**a**^

**Health behaviour**				
Intensive sports (hours per week)	1.27 (2.0)	1.68 (2.3)	0.85 (1.4)	**.018**^**a**^
Smoking behaviour				
Smokers [n (%)]	51 (29.3%)	25 (28.4%)	26 (30.2%)	.222 ^b^
Never smoked [n (%)]	61 (35.1%)	36 (40.9%)	25 (29.1%)	
Stopped smoking [n (%)]	62 (35.6%)	27 (30.7%)	35 (40.7%)	
				
Overweight (BMI)	28.00 (4.06)	27.84 (4.1)	28.17 (4.0)	.782 ^c^
Waist circumference	99.34 (11.0)	99.02 (11.1)	99.67 (10.9)	.696 ^a^
				
Sleeping behaviour				
No sleeping problems [n (%)]	91 (60.3%)	51 (65.4%)	40 (54.8%)	.386 ^b^
Some sleeping problems [n (%)]	43 (28.5%)	20 (25.6%)	23 (31.5%)	
Major sleeping problems [n (%)]	17 (11.3%)	7 (9.0%)	10 (13.7%)	

^+ ^= mean (SD), unless otherwise stated.

a = Mann Whitney U-test

b = Chi^2 ^test

c = t-test

**Table 3:** Parameters of chronic stress and job stress in the total sample and stratified for two groups of low vs. high stress reactivity ^+^

Characteristic	Total (n = 174)	Group with low stress reactivity (n = 88)	Group with high stress reactivity (n = 86)	P value
Chronic stress				
Total score SSCS	17.29 (7.2)	14.60 (6.1)	20.08 (7.2)	**< .001**^**c**^
				
Effort-reward imbalance				
Effort-reward ratio	0.77 (0.3)	0.70 (0.2)	0.84 (0.3)	**.000**^**a**^
Effort scale	14.84 (2.9)	14.05 (2.9)	15.65 (2.7)	**.000**^**c**^
Reward scale	44.56 (7.4)	46.22 (7.4)	42.86 (7.0)	**.003**^**c**^
Esteem scale	20.38 (3.8)	21.00 (3.8)	19.74 (3.7)	**.020**^**a**^
Job security scale	7.91 (2.2)	8.55 (2.0)	7.27 (2.3)	**.000**^**a**^
Job salary scale	16.26 (3.2)	16.67 (3.3)	15.85 (3.1)	**.023**^**a**^

^+ ^= mean (SD)

a = Mann Whitney U-test

b = Chi^2 ^test

c = t-test

**Table 4:** Biological stress parameters in the total sample and stratified for two groups of low vs. high stress reactivity ^+^

Characteristic	Total (n = 174)	Group with low stress reactivity (n = 88)	Group with high stress reactivity (n = 86)	P value
Allostatic Load Index	3.19 (2.19)	3.32 (2.2)	3.05 (2.1)	.484 ^a^

**Cardiovascular indicators**				
Blood pressure (diast.)	88.74 (9,6)	88.67 (9.8)	88.80 (9.4)	.928 ^c^
Blood pressure (systol.)	134.41 (14.2)	134.52 (13.9)	134,30 (14.6)	.919 ^c^
Hypertonus (>90/>140) [n (%)]	33 (19.0%)	18 (20.5%)	15 (17.4%)	.377 ^b^
Heart rate	76.55 (8.2)	76.95 (7.8)	76.15 (8.6)	.528 ^c^
Heart rate/night	63.77 (8.3)	63.66 (7.7)	63.89 (9.0)	855 ^c^

**Metabolic indicators**				
Total cholesterin	208.21 (34.1)	211.78 (33.0)	204.51 (34.9)	.164 ^c^
HDL	45.71 (9.5)	45.59 (9.5)	45.83 (9.5)	.865 ^c^
LDL	139.15 (29.1)	141.74 (29.4)	136.48 (28.65)	.238 ^c^

**Endocrine indicators**				
Cortisol				
Area under the curve morning	17.75 (6.1)	17.95 (6.23)	17.51 (5.9)	.746 ^c^
Area under the curve daytime	64.34 (24.4)	64.61 (23.7)	64.03 (25.3)	.855 ^c^
Increase morning cortisol	5.90 (6.0)	5.61 (5.9)	6.24 (6.1)	.358 ^c^
Increase daytime cortisol	-9,49 (37.9)	-6.30 (33.8)	-13.15 (42.1)	.855 ^c^
Daytime slope cortisol	- 0.58 (0.3)	- 0.56 (0.3)	- 0.59 (0.4)	.568 ^c^
				
Amylase				
Area under the curve morning	44.15 (51.8)	47.93 (66.1)	40.22 (30.5)	.746 ^c^
Area under the curve daytime	1155.98 (816.6)	1187.75 (894.0)	1124.61 (736.5)	.855 ^c^
Increase morning amylase	-12.21 (56.8)	-8.62 (71.6)	-15.94 (35.6)	.358 ^c^
Increase daytime amylase	-78.21 (529.7)	-84.45 (569.6)	-72.05 (490.5)	.222 ^c^
Daytime slope amylase	0.13 (0.2)	0.11 (0.2)	0.15 (0.231)	.222 ^c^

**Inflammatory indicators**				
Fibrinogen	331.84 (62.0)	334.06 (69.7)	329.55 (53.26)	.636 ^c^
CRP	0.20 (0.23)	0.19 (0.2)	0.21 (0.3)	.961^a^
Creatinin	1.02 (0.11)	1.03 (0.1)	1.01 (0.1)	.551 ^a^

^+ ^= mean (SD), unless otherwise stated.

a = Mann Whitney U-test

b = Chi^2 ^test

c = t-test

## Results

### Sample description

The average age of the study participants was 40.9 (7.8) years and 98.3% were male. Although participants have achieved a professional position with leadership responsibility, more than three fourths (76.9%) have a lower educational degree with less than 11 years of formal education. 23.6% achieved a master-level degree. For the total sample an average of 1.9 hours of unpaid overtime per month has been reported. 63% of all participants did shift work. Looking at the number of sick leave days per year, our sample is comparable to the total workforce of the company: 55.7% reported no sick leave days (versus 58.4% in the total company) and 12.6% were more than 10 days absent (versus 9.8% in the total company) (table 1). Due to legal and plant internal reasons, our assessment battery did not include objective figures on sick leave days and short-term disability.

### Comparison to the normal population

Compared to the normal population, our participants showed significantly poorer results in several parameters of psychosocial stress and in various health risk factors: The mean value for chronic stress (SSCS) was 17.29 in our sample compared to 14.37 for the reference population (p < .001; [39]). Looking at effort-reward imbalance (ERI), the mean value of 0.77 has been significantly higher than the reference value of 0.64 (p < .001; [40]). 5.7% of the total sample reported signs of clinically relevant anxiety (HADS > = 11), an additional 24.8% showed medium levels of anxiety (HADS > = 8 and < 11). Men in our sample had a significantly increased mean value of anxiety (6.02) versus the reference value for men (5.1; p = .048; [41]). 24.1% suffered from obesity (BMI > 30) versus a reference value of 14.1% (p < .001; [42]). The mean value for the stress reactivity summary score was 54.48 for the total group. 6.9% reached a value of above 70, the latter considered as critical value.

### Differences between employees with high and low stress reactivity

For the group with higher self-perceived stress reactivity a mean value of 63.21 (6.1) has been calculated. For the 50% with lower self-perceived stress reactivity the mean score was 45.95 (5.2). For demographic and professional variables, significant differences between high and low stress-reactive employees were found only for the educational background of the subjects, with more persons with master-level degree in the low stress-reactive group (see table 1).

High vs. low stress-reactive employees differed significantly in the extent of health-related complaints (GBB-24), especially with respect to musculoskeletal and cardiovascular complaints. Also for depression and anxiety we found poorer results for high stress-reactive persons. Looking at health behaviour the two groups showed a significant difference for intensive sports activity (table 2).

As expected, groups of high stress-reactive versus low stress-reactive employees differed significantly in several psychosocial stress parameters: chronic stress (SSCS) and all ERI-scales (table 3).

High vs. low stress-reactive employees did not show significant differences in any specific biological stress indicators, nor in the revised global allostatic load index. The mean allostatic load value was 3.32 (SD 2.2) for the high stress-reactive group and 3.05 (SD 2.1) for the low stress-reactive group (p = .484) (table 4).

### Prediction of self-perceived stress reactivity

Logistic regression model with self-perceived stress reactivity as dependent variable revealed significant predictive power of education, cardiovascular complaints, chronic stress, and effort-reward imbalance. Thus the results of the univariate analyses were confirmed by the regression model. The most significant effect to predict self-perceived stress was found for education: Compared to the employees with master degree, persons with less years of formal education (school until age 17) showed a 13-fold (95% CI 3.6-46.6) and those with the lowest educational level (school until age 15) a 6.3-fold (95% CI 2.2-17.8) higher risk for high stress reactivity. The risk of employees in the highest tertile of cardiovascular complaints was 5.1-fold higher (95% CI 2.0-13.0) compared to those with low level cardiovascular complaints. Self-assessed increased chronic stress was also associated with increased self-perceived stress reactivity. As to the effort-reward ratio, employees with moderate values in this scale are found at the highest risk for high stress reactivity, and not those with the worst imbalance.

Equally examined in the model were the effects of esteem, security, and promotion (ERI subscales), gastrointestinal complaints and musculoskeletal complaints (GBB-24), HADS anxiety and depression, intensive sports, smoking, total cholesterol, and daytime slope amylase; they did not significantly change the prediction and were thus removed by the stepwise procedure.

## Discussion

Referring to the main hypothesis (H1), our study shows that in industrial employees holding a middle management ("sandwich") position in manufacturing, increased individual stress reactivity is indeed associated with heightened psychosocial work stress, a higher rate of psychosomatic and physical complaints, poorer psychological health, and negative health behaviour. In addition, high stress-reactive employees experience an increased effort-reward imbalance (especially less job security and less esteem), show higher levels of depression and anxiety, and suffer more often from musculoskeletal and cardiovascular complaints. Compared to the normal population, our industrial workplace sample showed poorer results in terms of self-rated chronic stress, effort-reward ratio, anxiety, a variety of other related psychological problems, and obesity. These findings are of considerable interest since a study on work-related stress in a population of employed Swedish women showed an association between work-related stress, illness symptoms and sick-leave [43]. Furthermore a recent meta-analysis [44] reported psychosomatic complaints and/or suffering from psychological problems as significant predictors to sickness absence. Our findings may reflect the overall growing economical and organisational pressure of increased productivity which our sample is dealing with. Indeed, like in many other industries and places worldwide, production in our sample increased by 20% between 2003 and 2007 while keeping the workforce numbers nearly at the same level.

In respect to H2, our data revealed that education, cardiovascular complaints, chronic stress, and effort-reward imbalance were moderate predictors for perceived stress reactivity. Contrary to our expectations and to the few findings available of somatic correlates of stress phenomena in working place samples [26,45], we did not find any relationship between perceived stress reactivity on one hand and specific biological indicators of stress or generic measures of somatic stress reaction, e.g. allostatic load index, on the other. Thus, our preliminary findings do not support data which suggest that prolonged psychosocial job stress [14] affects the biological stress axis and may consecutively cause severe illnesses and negative health outcomes. The following reasons may explain our results:

1. Unlike the only workplace study investigating the interaction of job profile and allostatic load [26], our participants were selected primarily from a middle management ("sandwich") position within the production line of an international manufacturer. Better work position and specific job demands of our middle-aged target group may have led to better health and coping capabilities in comparison to a sample of predominantly blue collar workers. 2. Especially with regard to biological parameters, a healthy worker effect may be assumed i.e., those in employment are known to be healthier than those not working [46].

Some more general factors may also explain our cross sectional finding: lack of longitudinal data, age of the participants, and a selection bias due to voluntary participation in the study.

We do recognise that there are some additional limitations of our study. First, our results cannot be generalised to other working populations. As participation was voluntary it can be assumed that all participants in the current study were in at least some positive stage of change [47]. Based on the clinical impression due to the interviews at baseline, a selection bias can be assumed because most of the participants were highly self-motivated with respect to their attitude towards demands of work and health. Second, as self-reported data on stress may lead to underestimation or overestimation of the real work stress situation, we are not able to objectively describe the work load of our sample. Third, our study faces a well known dilemma in stress research of bridging the gap between biological and psychosocial concepts and methods of stress assessment. The dilemma becomes even clearer as there is still a lack of standardised norm and cut-off values for job stress. However, here we present cross sectional findings only, but especially longitudinal data might be useful for a better understanding of the relationship and pathways between psychosocial and biological stress parameters and their impact on disability, diseases, and workers' productivity.

Finally, we conclude that the concept of stress reactivity as measured by the SRS correlates with various important aspects of psychosocial impairment and maladaptive health behaviour and thus indeed may be a valid tool to identify employees potentially prone to suffer from chronic job stress.

## Conclusions

The results of this cross sectional study in a sample of middle management employees in the production line of an international plant demonstrate that in a relevant subgroup of the whole sample psychosocial stress parameters and well known health risk factors are significantly increased. Self-report data on stress reactivity allow to identify employees who are under stress according to established models and who suffer from stress-associated complaints. They may thus be at risk for severe stress sequelae. However, this cross sectional study failed to demonstrate a significant relationship between psychosocial and biological stress indicators. Longitudinal data may further elucidate the pathways of perceived stress and health status, and thus may prove if the measurement of dispositional stress reactivity may indeed serve as a predictor for adverse long term stress-related health outcomes.

## Competing interests

The authors declare that they have no competing interests.

## Authors' contributions

HL, PA, MH, BMM, UN and HG made substantial 1) contributions to conception and design, acquisition, analysis and interpretation of data 2) have involved in drafting the manuscript.

All authors read and approved the final manuscript.

## Pre-publication history

The pre-publication history for this paper can be accessed here:

http://www.biomedcentral.com/1471-2458/10/252/prepub
